# Efficacy and Safety of Adjunctive Corticosteroids in Children With Kawasaki Disease: A Systematic Review and Meta-Analysis of Randomized Trials

**DOI:** 10.14740/cr2300

**Published:** 2026-10-24

**Authors:** Niemat Mohammed Tahir Ali, Ahmad Ayed AlShammari, Rana Alshehri, Renas Muneef Ageeli, Mohammed Hasen Mohammed Alghamdi, Khadijah Jadah Mohammed Asiri, Refad Talal AlKaki, Narmen Hamadelnil Ibrahim Eltrafi, Norah Ali Alwarthan, Wejdan Abdullah Omar Bamusa, Nourah AlNamash, Fahad Juwayid H. Alqahtani, Lara Sadik, Ahmed Sobhy

**Affiliations:** a Department of Paediatrics and Child Health, College of Medicine, King Khalid University, Abha, Saudi Arabia; b Department of Pediatrics, College of Medicine, Imam Abdulrahman Bin Faisal University, Dammam, Saudi Arabia; c Jeddah University, Jeddah, Saudi Arabia; d College of Medicine, Jazan University, Jazan, Saudi Arabia; e Ministry of Health, Abha, Aseer, Saudi Arabia; f Saudi German Hospital Aseer, Khamis Mushait, Saudi Arabia; g Alrayan Colleges, Medina, Saudi Arabia; h Mediclinic Airport Road Hospital, Abu Dhabi, United Arab Emirates; i Imam Mohammad Ibn Saud Islamic University, Riyadh, Saudi Arabia; j Unaffiliated, Jeddah, Saudi Arabia; k Farwaniya Hospital, Ministry of Health, Kuwait City, Kuwait; l Ministry of Health, Riyadh, Saudi Arabia; m College of Medicine, Alfaisal University, Riyadh, Saudi Arabia; n Faculty of Medicine, Kafr Elsheikh University, Kafr Elsheikh, Egypt

**Keywords:** Kawasaki disease, Corticosteroids, Prednisolone, Methylprednisolone, Intravenous immunoglobulin, C-reactive protein, Coronary artery lesions, Meta-analysis

## Abstract

**Background:**

Kawasaki disease is an acute pediatric vasculitis and a major cause of acquired heart disease in children. Although intravenous immunoglobulin (IVIG) with aspirin remains the standard treatment, some patients have persistent inflammation, IVIG resistance, or coronary complications. The role of adjunctive corticosteroids remains debated, particularly across different risk populations and corticosteroid regimens. This systematic review and meta-analysis evaluated the efficacy and safety of adjunctive corticosteroid therapy in children with Kawasaki disease.

**Methods:**

A systematic search of PubMed, Scopus, Cochrane CENTRAL, and Web of Science was conducted to identify randomized controlled trials comparing corticosteroid-containing treatment strategies with non-corticosteroid comparators, including standard IVIG-based initial treatment or additional IVIG in IVIG-resistant disease. The primary outcome was the change in C-reactive protein levels. Secondary outcomes included fever duration, IVIG resistance, laboratory markers, coronary outcomes, and adverse events. Pooled estimates were calculated using random-effects models and reported as mean differences (MD) or risk ratios with 95% confidence intervals (CIs).

**Results:**

Ten randomized controlled trials, including 5,796 unique children, were included. Adjunctive corticosteroid therapy was associated with a greater reduction in C-reactive protein levels compared with usual care alone (MD, −11.05; 95% CI, −18.27 to −3.84; P = 0.0027; I^2^ = 34.3%, P = 0.179). Corticosteroid therapy also reduced fever duration (MD, −1.03; 95% CI, −1.70 to −0.36; P = 0.0025; I^2^ = 85.2%, P < 0.001) and white blood cell count (MD, −1.07; 95% CI, −1.98 to −0.17; P = 0.020; I^2^ = 0.0%, P = 0.887). No significant differences were observed in IVIG resistance, coronary artery lesions, large/giant aneurysms, any adverse event, or adverse events leading to discontinuation.

**Conclusions:**

Adjunctive corticosteroids were associated with improved inflammatory and early clinical response in Kawasaki disease, mainly through greater C-reactive protein reduction and shorter fever duration. However, corticosteroids did not significantly reduce overall coronary outcomes, and no statistically significant increase in the pooled adverse-event outcomes was detected. These findings support selective, risk-based use rather than routine universal administration.

## Introduction

Kawasaki disease is an acute systemic vasculitis of unknown etiology that primarily affects infants and young children, particularly those younger than 5 years [1]. Although Kawasaki disease is reported worldwide, it remains most prevalent in Asia and represents a major cause of acquired heart disease among children [2]. Its clinical significance is largely driven by coronary artery involvement, as coronary artery lesions may progress to aneurysm formation, occlusion, myocardial infarction, or death [3]. Coronary artery disease in Kawasaki disease is thought to reflect immune-mediated vascular injury, with cytokine cascade activation and endothelial cell activation contributing to arterial inflammation [4].

The standard treatment for acute Kawasaki disease consists of high-dose intravenous immunoglobulin (IVIG) combined with aspirin [1]. This regimen reduces systemic inflammation and lowers the occurrence of coronary artery abnormalities, but it does not eliminate the risk of adverse coronary outcomes [5]. Coronary artery lesions still develop in approximately 10% to 20% of treated patients, and 8% to 25% develop resistance to IVIG, which is itself a risk factor for coronary artery lesions [6–8]. Accordingly, improved adjunctive strategies are needed, particularly for patients with persistent inflammation or predicted poor response to standard therapy.

Corticosteroids have been proposed as adjunctive therapy because Kawasaki disease is characterized by marked immune activation and elevated inflammatory cytokines [9, 10]. Reducing circulating cytokine levels is a plausible therapeutic goal, as these mediators may contribute to endothelial injury, coronary artery disease, and systemic inflammatory manifestations [11]. Previous reports suggest that corticosteroids may hasten the resolution of inflammation and improve clinical recovery, although their role in Kawasaki disease has remained controversial [12, 13].

Randomized evidence has been inconsistent across populations and corticosteroid regimens [14, 15]. The RAISE trial in high-risk Japanese patients showed that adjunctive longer-course prednisolone reduced coronary artery lesions, whereas the North American trial in unselected patients found no coronary benefit from single-pulse intravenous methylprednisolone [15, 16]. Most recently, Lin et al [14] reported that adjunctive prednisolone did not reduce coronary artery lesions at 1 month in an unselected Kawasaki disease population, reinforcing uncertainty regarding routine use across broader clinical settings.

Therefore, this systematic review and meta-analysis aimed to evaluate the efficacy and safety of adjunctive corticosteroid therapy compared with usual care in children with Kawasaki disease, focusing on coronary outcomes, treatment response, inflammatory markers, and adverse events.

## Materials and Methods

### Study design and reporting

This systematic investigation was executed following the methodological recommendations of the Cochrane Handbook and reported according to the reporting standards of the Preferred Reporting Items for Systematic Reviews and Meta-Analyses (PRISMA) statement [17, 18]. The planned protocol was prospectively registered on PROSPERO (CRD420261444980), specifying randomized controlled trials (RCTs) as the primary evidence base and defining the change in C-reactive protein (CRP) levels as the primary outcome, with clinical, inflammatory, and safety outcomes assessed as secondary endpoints. CRP was selected a priori as the primary outcome because it was expected to provide a consistently reported quantitative measure of the early systemic inflammatory response across randomized trials. This choice was prespecified in the registered protocol and was not based on the direction or statistical significance of the subsequently observed results.

### Eligibility criteria

Eligible studies were RCTs enrolling pediatric patients, aged 18 years or younger, with a diagnosis of Kawasaki disease. Studies were eligible if they compared a corticosteroid-containing treatment strategy, including prednisolone or methylprednisolone, with a non-corticosteroid comparator. In trials evaluating initial treatment, the comparator generally consisted of standard IVIG-based therapy with aspirin. In trials enrolling patients with established IVIG-resistant Kawasaki disease, an additional dose of IVIG was considered an eligible active rescue comparator. These rescue-treatment comparisons were considered clinically distinct from trials evaluating corticosteroids during initial therapy.

Studies were required to report at least one relevant efficacy or safety outcome, including coronary artery abnormalities or lesions, fever duration, IVIG resistance, inflammatory markers, laboratory parameters, or adverse events. Trials including general Kawasaki disease, high-risk Kawasaki disease, or IVIG-resistant Kawasaki disease populations were considered eligible. Non-randomized studies, observational studies, case reports, reviews, editorials, animal studies, laboratory studies, and reports with insufficient or non-extractable outcome data were excluded.

### Literature search and study selection

A systematic literature search was performed from database inception to May 2026 using PubMed, Scopus, Web of Science, and Cochrane CENTRAL. The search strategy combined terms related to Kawasaki disease, corticosteroid therapy, prednisolone, methylprednisolone, steroids, and RCTs. The database-specific search strategy for all databases is presented here (Supplementary Material 1, cr.elmerpub.com).

After removal of duplicate records, two reviewers (AAA and RA) independently screened titles and abstracts for eligibility. Potentially relevant reports underwent full-text assessment by these two reviewers. Disagreements were resolved through discussion, and a third reviewer (AS) was consulted when consensus could not be reached. Reference lists of included studies and relevant reviews were also screened to identify additional eligible reports. At the full-text assessment stage, each excluded report was assigned one principal reason for exclusion to avoid double counting. Reasons were categorized as ineligible study design, ineligible population, ineligible intervention or comparator, absence of eligible or extractable outcome data, duplicate or overlapping publication, or other eligibility-related reasons.

### Outcomes

The primary outcome was the change in CRP levels, assessed as the difference in CRP reduction between adjunctive corticosteroid therapy plus usual care and usual care alone. CRP was treated as a surrogate marker of inflammatory response rather than as a direct measure of clinical or coronary benefit. Coronary artery outcomes and IVIG resistance were therefore analyzed separately as key clinical outcomes, and no assumption was made that a greater reduction in CRP necessarily translated into improved coronary outcomes. When trials reported CRP using comparable continuous measures, data were pooled using mean differences (MDs) with 95% confidence intervals (CIs).

Secondary outcomes included clinical response, laboratory response, coronary outcomes, and safety endpoints. Clinical response outcomes included duration of fever and IVIG resistance. Laboratory outcomes included serum albumin, white blood cell count, and platelet count. Coronary outcomes included coronary artery lesions and large or giant coronary aneurysms. Safety outcomes included any adverse event, serious adverse events, adverse events leading to treatment discontinuation or dose modification, and specific treatment-emergent adverse effects reported by the individual trials. Specific adverse effects were summarized descriptively because their definitions, ascertainment, and reporting differed substantially among studies.

Outcome definitions were accepted as reported by the original trials. For IVIG resistance, definitions based on persistent or recurrent fever after completion of initial IVIG therapy were considered eligible. Coronary outcomes were pooled when trial definitions were clinically comparable, including coronary artery lesions, coronary artery abnormalities, or aneurysm-related outcomes. Where outcome definitions, measurement timing, or reporting formats differed substantially, findings were synthesized only when appropriate and interpreted with consideration of clinical and methodological heterogeneity.

### Data extraction

Two reviewers (AAA and RA) independently extracted data using a standardized data extraction form. Extracted information included study ID, arms, country, study design, trial or center name, recruitment period, population type, baseline coronary artery status, definition of IVIG resistance, follow-up duration, inclusion criteria, sample size, intervention and comparator details, and reported outcomes. For safety outcomes, the definitions and ascertainment methods reported by each trial were also extracted and are detailed (Supplementary Material 2, cr.elmerpub.com).

Baseline data were extracted when available, including age, sex distribution, CRP, white blood cell count, platelet count, erythrocyte sedimentation rate, albumin, and coronary artery z-scores. For dichotomous outcomes, the number of events and total participants in each group were extracted. For continuous outcomes, means, standard deviations, and sample sizes were extracted.

For clinical interpretation, trials were categorized as initial therapy in unselected patients, intensified initial therapy in predicted high-risk patients, or rescue therapy in established IVIG-resistant disease.

### Risk of bias assessment

Risk of bias was assessed using the Cochrane Risk of Bias 2 tool (RoB 2) for RCTs [19]. The assessed domains included bias arising from the randomization process, bias due to deviations from intended interventions, bias due to missing outcome data, bias in measurement of the outcome, and bias in selection of the reported result. Each domain was judged as low risk, some concerns, or high risk of bias, followed by an overall risk-of-bias judgment for each study. Disagreements between reviewers were resolved by consensus or consultation with a third reviewer (AS).

### Statistical analysis

Meta-analysis was performed using a random-effects model to account for expected clinical and methodological variability across trials. Risk ratios (RRs) with 95% CIs were used for dichotomous outcomes, including coronary artery lesions, large or giant aneurysms, IVIG resistance, and adverse events. Safety outcomes were quantitatively pooled only when the reported endpoint was sufficiently comparable across trials. Trial-specific adverse effects and safety definitions that could not be harmonized were summarized descriptively. MDs with 95% CIs were used for continuous outcomes, including fever duration, CRP reduction, serum albumin, white blood cell count, and platelet count.

Between-study heterogeneity was assessed using Cochran’s Q test and the I^2^ statistic. Heterogeneity was interpreted according to the magnitude of I^2^ and the statistical significance of the Q test. A P value less than 0.05 was considered statistically significant for pooled treatment effects. Sensitivity analysis was performed using leave-one-out analysis to evaluate whether the overall pooled estimate was driven by any single study. Galbraith plots were used to identify studies contributing to between-study variability. Meta-regression was performed when data were available to explore whether study-level baseline characteristics, including mean age and male proportion, explained variability in treatment effects.

Publication bias and small-study effects were assessed using the Doi plot and Luis Furuya-Kanamori (LFK) index. The Doi plot was used as a visual method to evaluate asymmetry in the distribution of study effects, while the LFK index was used to quantify the degree of asymmetry. The LFK index was interpreted using standard thresholds: values within ± 1 were considered to indicate no asymmetry, values between ± 1 and ± 2 suggested minor asymmetry, and values beyond ± 2 indicated major asymmetry. In addition, a conventional funnel plot was constructed for the primary outcome (change in CRP levels) to provide a visual assessment of potential small-study effects and publication bias. Because fewer than 10 studies contributed to this analysis, interpretation of funnel-plot asymmetry was considered exploratory.

### Ethical compliance

No new human-subject research was conducted. This systematic review and meta-analysis was conducted using previously published aggregate trial data and involved no direct interaction with human participants or collection of identifiable individual patient information. The study was conducted and reported according to recognized standards for ethical and transparent secondary research. Consequently, new institutional ethics committee approval and individual informed consent were not required for this secondary analysis.

## Results

### Databases search

The database search identified 2,151 records, including 653 from PubMed, 925 from Scopus, 54 from Cochrane CENTRAL, and 519 from Web of Science. After removal of 754 duplicate records, 1,397 records underwent title and abstract screening. Of these, 1,032 records were excluded because they clearly did not meet the prespecified eligibility criteria. The full texts of 365 potentially eligible reports were subsequently assessed. A total of 355 reports were excluded after full-text review because of ineligible study design, ineligible population, ineligible intervention or comparator, absence of eligible or extractable outcome data, duplicate or overlapping publication, or other eligibility-related reasons. Ten eligible reports were retained for inclusion in the systematic review and synthesis [12–16, 20–24] (Fig. 1).

**Figure 1:**
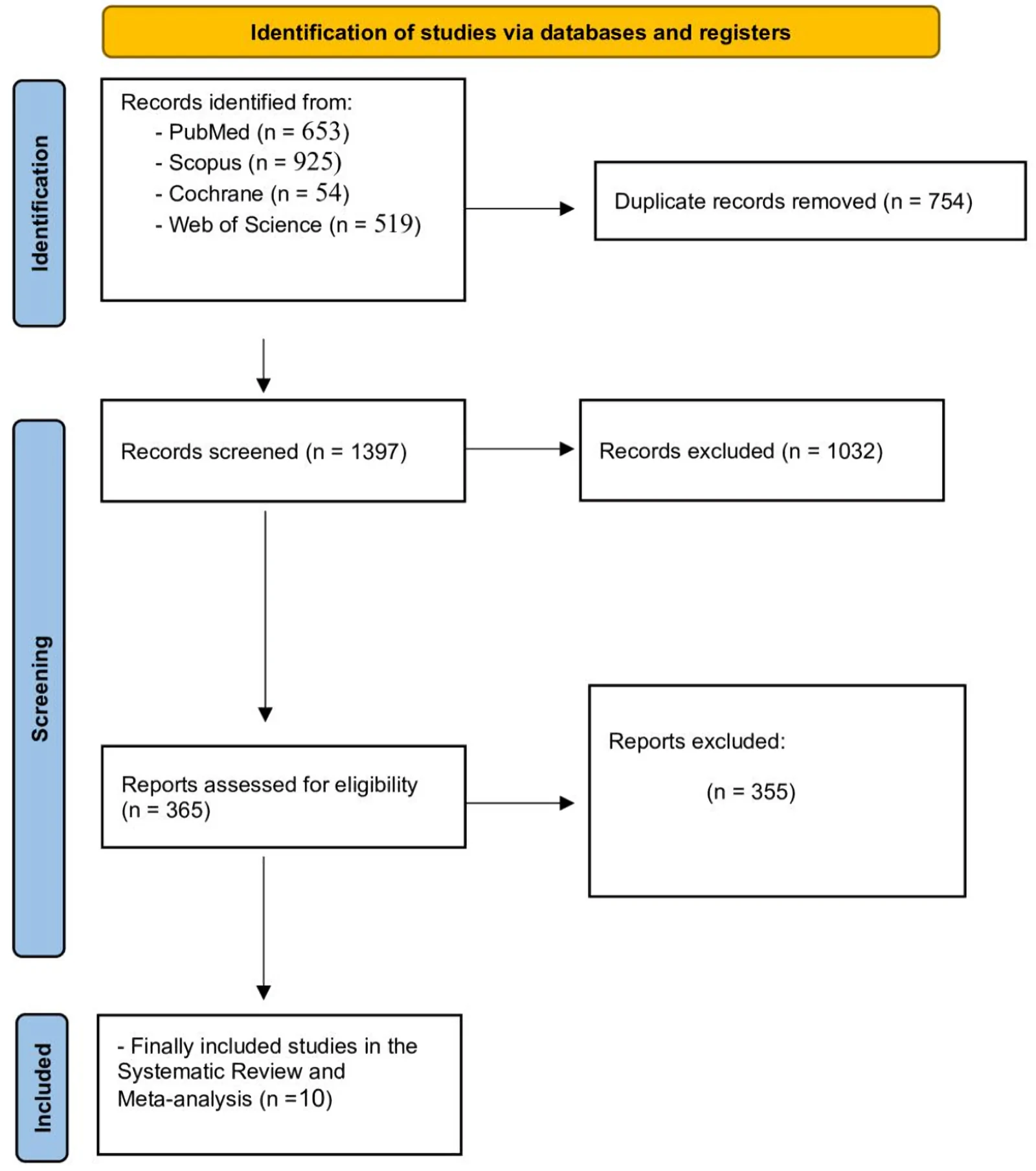
PRISMA flow diagram of study selection. PRISMA: Preferred Reporting Items for Systematic Reviews and Meta-Analyses.

### Characteristics of the included studies

The 10 included studies were RCTs enrolling 5,796 children with Kawasaki disease, with sample sizes ranging from 15 to 3,208 participants. Studies were conducted in China, Japan, North America, and the United States, with recruitment periods spanning from 1998 to 2024. The included trials evaluated corticosteroid-containing treatment strategies across different clinical settings. Most trials assessed corticosteroids during initial treatment and used standard IVIG-based therapy as the comparator. In contrast, the trials by Miura et al in 2005 [20] and 2008 [21], and Wang et al [24] enrolled patients with established IVIG-resistant Kawasaki disease and directly compared methylprednisolone pulse therapy with an additional dose of IVIG as rescue treatment. The study populations included general or unselected Kawasaki disease, high-risk Kawasaki disease, and IVIG-resistant Kawasaki disease. Baseline coronary artery status varied across studies; some trials excluded patients with baseline coronary artery abnormalities, whereas others included mixed populations with and without baseline coronary involvement. Follow-up duration ranged from 0.25 to 50 months. The majority of participants were young children with a mean age ranging from 1.8 to 5.7 years. Within individual trial arms, the proportion of male participants generally ranged from 50.0% to 71.4%. Baseline inflammatory markers and coronary artery z-scores were variably reported but were generally comparable between treatment groups within individual studies. Accordingly, additional IVIG was represented within the randomized evidence as an active rescue comparator rather than being excluded from consideration. Evidence from these rescue trials was interpreted separately from the clinical question of corticosteroid use during initial treatment. The included trials represented distinct clinical settings: unselected initial therapy, intensified initial therapy in predicted high-risk patients, and rescue treatment after established IVIG resistance. These settings were interpreted separately because they address different therapeutic questions. Detailed information about the summary and baseline characteristics is presented in Table 1 [12–16, 20–24] and Supplementary Material 3 (cr.elmerpub.com).

**Table 1:** Baseline Characteristics of Included Studies

Study	Arms	Sample size	Age, mean (SD)	Male, n (%)	C-reactive protein (mg/L), mean (SD)	White blood cell count (× 10^3^/µL), mean (SD)	Platelet count (× 10^4^/µL), mean (SD)	ESR (mm/h), mean (SD)	Albumin (g/dL), mean (SD)	LAD z-score, mean (SD)	LAD z-score, mean (SD)	LMCA z-score, mean (SD)
Lin et al, 2026 [14]	Standard Tx + corticosteroids	1,604	2.7 (1.8)	973 (60.7)	NA	NA	NA	NA	NA	1.1 (1.2)	0.8 (1.1)	0.9 (1.2)
	Standard Tx	1,604	2.7 (1.9)	967 (60.3)	NA	NA	NA	NA	NA	1 (1.2)	0.8 (1.1)	0.8 (1.1)
Kobayashi et al, 2012 [15]	Standard Tx + corticosteroids	121	2.6 (2.3)	67 (55.4)	93 (54.1)	15.4 (4.9)	27.4 (8.1)	NA	NA	1.6 (0.8)	1.4 (0.9)	1.6 (0.6)
	Standard Tx	121	2.5 (2.1)	68 (56.2)	88 (42.2)	15.6 (4.7)	28.9 (8.9)	NA	NA	1.7 (0.6)	1.6 (0.9)	1.7 (0.6)
Ogata et al, 2012 [22]	Standard Tx + corticosteroids	22	3.4 (0.1)	11 (50)	87 (25.9)	13.5 (2.9)	26.8 (3.3)	NA	3.7 (0.2)	1.4 (1.3)	1.5 (1.2)	1.1 (1.4)
	Standard Tx	26	3.5 (0.4)	13 (50)	98 (25.9)	15 (3.6)	31.5 (6.7)	NA	3.7 (0.3)	1.5 (1.2)	2.2 (1.3)	1.3 (1.5)
Newburger et al, 2007 [16]	Standard Tx + corticosteroids	101	2.9 (2.7)	63 (62.4)	72 (79.3)	13.5 (4.6)	39.4 (13.8)	65.5 (33.3)	3.3 (0.6)	1.1 (1.6)	1.1 (1.5)	0.8 (1.0)
	Standard Tx	98	2.9 (2.1)	61 (62.2)	68 (108.9)	12.9 (5.3)	39.3 (14.7)	67.0 (39.3)	3.3 (0.6)	1.1 (1.5)	1.2 (1.5)	0.9 (1.2)
Inoue et al, 2006 [12]	Standard Tx + corticosteroids	90	2.4 (1.9)	51 (56.7)	89 (49)	13.9 (3.9)	35.1 (9.2)	NA	4 (0.4)	NA	NA	NA
	Standard Tx	88	2.3 (1.7)	51 (58)	91 (51)	14.7 (4.5)	35.8 (9.9)	NA	4 (0.4)	NA	NA	NA
Sundel et al, 2003 [23]	Standard Tx + corticosteroids	18	5.7 (3.3)	12 (66.7)	9.2 (4.6)	13.6 (4.8)	40.1 (13.6)	74.5 (25.6)	3.2 (0.4)	NA	NA	NA
	Standard Tx	21	5.4 (2.9)	15 (71.4)	11.9 (5.9)	13.7 (4.9)	35 (11)	77 (23.6)	2.9 (0.6)	NA	NA	NA
Wang et al, 2020 [24]	Standard Tx + corticosteroids	40	2.1 (1.8)	28 (70)	123.1 (69.8)	18.7 (6.9)	40.8 (17.7)	NA	9.5 (1.3)	NA	NA	NA
	Standard Tx	40	1.8 (1.2)	28 (70)	100.4 (56.3)	16.1 (6.9)	44.7 (21.1)	NA	9.3 (1.1)	NA	NA	NA
Miura et al, 2008 [21]	Standard Tx + corticosteroids	7	2.7 (1.6)	5 (71.4)	80 (70)	12.6 (4.8)	NA	NA	NA	NA	NA	NA
	Standard Tx	8	2.6 (2.2)	5 (62.5)	56 (48)	15.1 (4.9)	NA	NA	NA	NA	NA	NA
Miura et al, 2005 [20]	Standard Tx + corticosteroids	11	NA	NA	NA	NA	NA	NA	NA	NA	NA	NA
	Standard Tx	11	NA	NA	NA	NA	NA	NA	NA	NA	NA	NA
Okada et al, 2003 [13]	Standard Tx + corticosteroids	14	3.1 (2.3)	8 (57.1)	NA	NA	NA	NA	NA	NA	NA	NA
	Standard Tx	18	2.4 (1.9)	10 (55.6)	NA	NA	NA	NA	NA	NA	NA	NA

Tx: treatment; SD: standard deviation; CRP: C-reactive protein; WBC: white blood cell count; ESR: erythrocyte sedimentation rate; LAD z-score: left anterior descending coronary artery z-score; LMCA z-score: left main coronary artery z-score; NA: not available; ; Albumin: serum albumin; Male n (%): number and percentage of male participants.

### Risk of bias

Risk of bias was assessed using the Cochrane RoB 2 tool across five domains: randomization process, deviations from intended interventions, missing outcome data, outcome measurement, and selection of reported results. Overall, all included trials were judged to have a low risk of bias. Most studies showed low risk across all assessed domains. However, Sundel et al 2003 [23] and Okada et al 2003 [13] had some concerns in the selection of the reported result domain, while Miura et al 2008 [21] had some concerns in outcome measurement. No study was judged to have high risk of bias. Overall, the risk-of-bias assessment supports the methodological reliability of the included randomized evidence, although a few domain-specific concerns should be considered when interpreting individual trial findings (Fig. 2).

**Figure 2:**
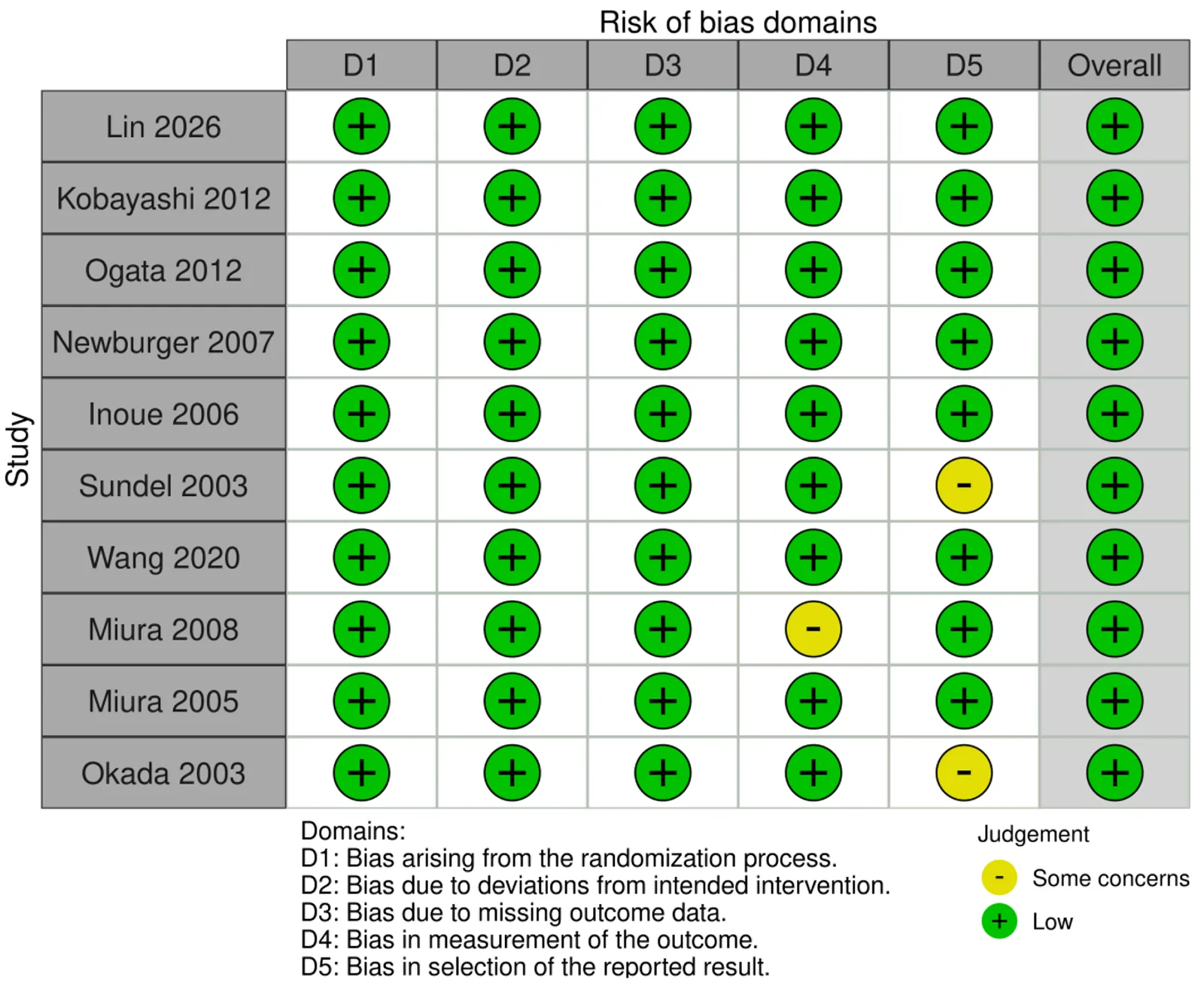
Risk-of-bias assessment of the included randomized controlled trials.

### Change in CRP levels

Six studies reported changes in CRP levels, including 1,895 patients receiving adjunctive corticosteroid therapy plus usual care and 1,897 receiving usual care. Adjunctive corticosteroid therapy plus usual care was associated with a greater reduction in CRP levels compared with usual care alone (MD, −11.05; 95% CI, −18.27 to −3.84; P = 0.0027). Heterogeneity was moderate and not statistically significant (I^2^ = 34.3%, P = 0.179) (Fig. 3). This finding reflects a greater early anti-inflammatory response and should not be interpreted as evidence of coronary protection.

**Figure 3:**
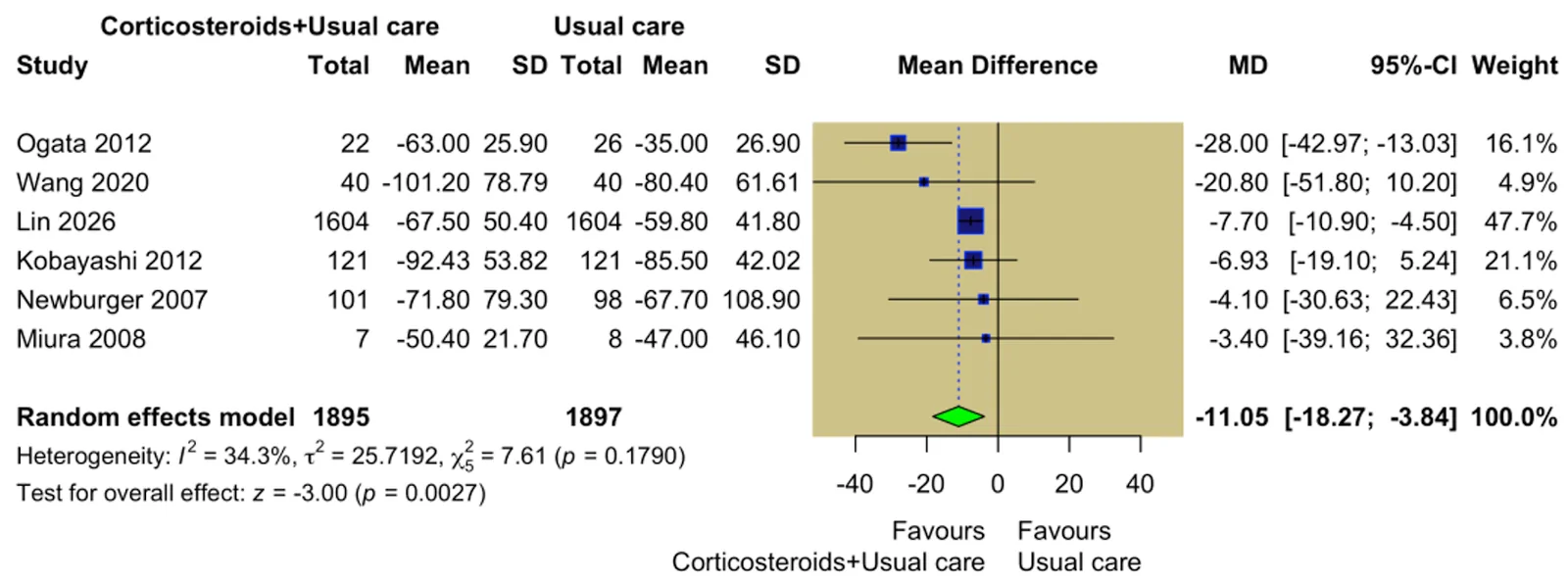
Forest plot of change in C-reactive protein levels with adjunctive corticosteroid therapy versus usual care. CI: confidence interval; MD: mean differences; SD: standard deviation.

Leave-one-out sensitivity analysis showed that the direction and statistical significance of the pooled estimate were maintained after omitting each study individually. The pooled MDs ranged from −14.07 after omitting Lin et al 2026 [14] to −7.70 after omitting Ogata et al 2012 [22], indicating that no single study fully explained the overall association (Fig. 4). The Galbraith plot identified two studies outside the 95% confidence region, suggesting that these studies may have contributed to between-study variability (Supplementary Material 4, cr.elmerpub.com).

**Figure 4:**
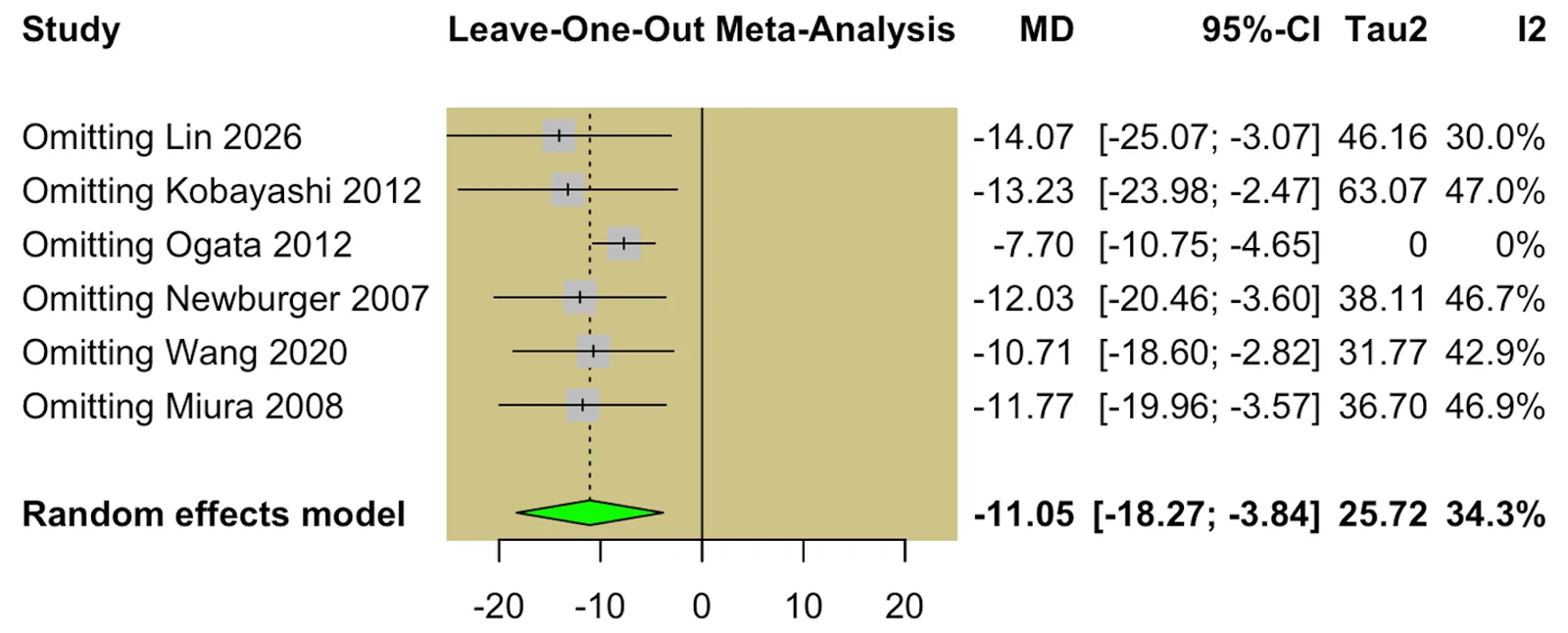
Leave-one-out sensitivity analysis for change in C-reactive protein levels. CI: confidence interval; MD: mean differences; SD: standard deviation.

Meta-regression was performed to explore whether baseline age or male proportion explained variability in the pooled effect for CRP reduction. In the age-based model, increasing mean age showed a borderline inverse association with the magnitude of CRP reduction (β = −17.11; 95% CI, −34.51 to 0.28; P = 0.054). Residual heterogeneity was not significant (I^2^ = 0.0%; QE P = 0.421), and the model accounted for the observed between-study heterogeneity (R^2^ = 100.0%) (Supplementary Material 5, cr.elmerpub.com).

Similarly, male proportion showed a borderline association with the treatment effect (β = 1.06; 95% CI, −0.11 to 2.22; P = 0.076). Residual heterogeneity was not significant (I^2^ = 0.0%; QE P = 0.347), with the model accounting for the observed heterogeneity (R^2^ = 100.0%) (Supplementary Material 6, cr.elmerpub.com).

The Doi plot showed no asymmetry (LFK index = −0.84); however, interpretation was limited by the small number of studies (Supplementary Material 7, cr.elmerpub.com). The conventional funnel plot appeared visually asymmetric, whereas the Doi plot LFK index did not indicate asymmetry; neither finding was considered conclusive given the small number of included studies (n = 6) (Supplementary Material 8, cr.elmerpub.com).

### Clinical response and treatment failure outcomes

The pooled estimate of 571 participants revealed a statistically significant reduction in fever with adjunctive corticosteroid therapy compared with usual care alone (MD, −1.03; 95% CI, −1.70 to −0.36; P = 0.0025). Heterogeneity remained substantial (I^2^ = 85.2%; P < 0.001) (Fig. 5).

**Figure 5:**
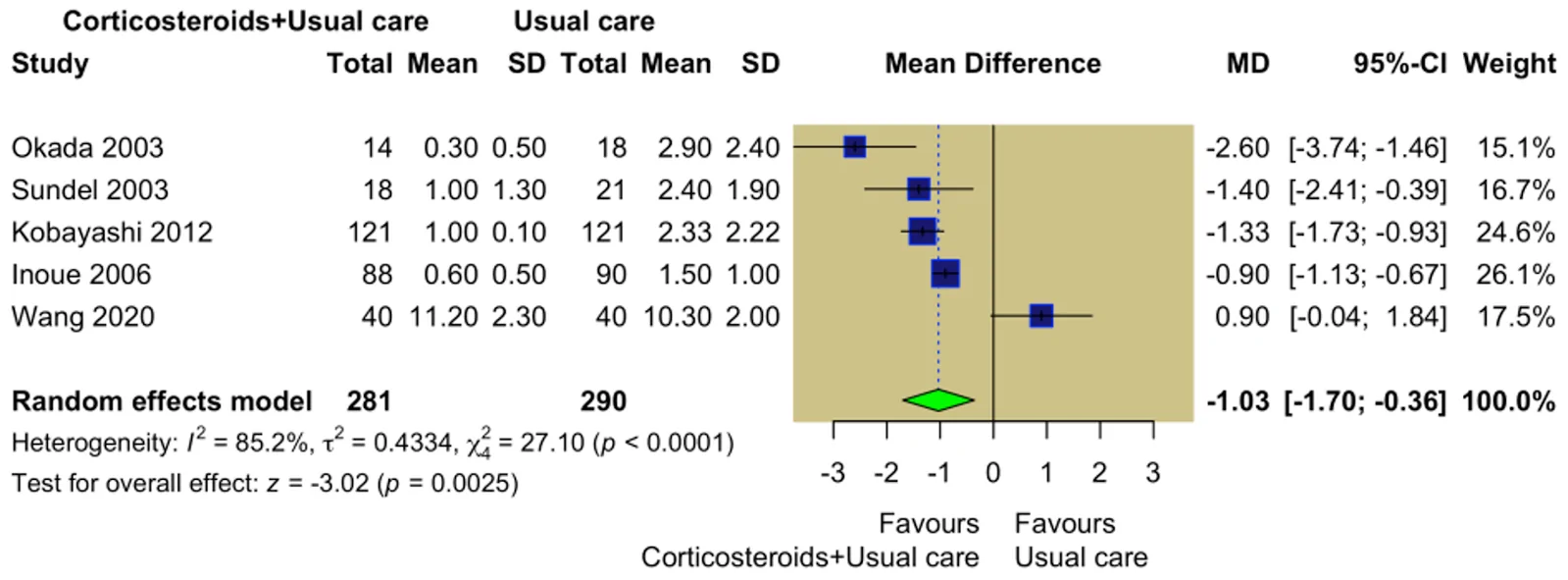
Forest plot of fever duration with adjunctive corticosteroid therapy versus usual care. CI: confidence interval; MD: mean differences; SD: standard deviation.

Five studies assessed IVIG resistance, including 3,875 participants. Adjunctive corticosteroid therapy was associated with a lower risk of IVIG resistance compared with usual care alone, but the pooled estimate was not statistically significant (RR, 0.57; 95% CI, 0.30 to 1.08; P = 0.083). Heterogeneity was substantial (I^2^ = 82.3%, P = 0.0001) (Fig. 6).

**Figure 6:**
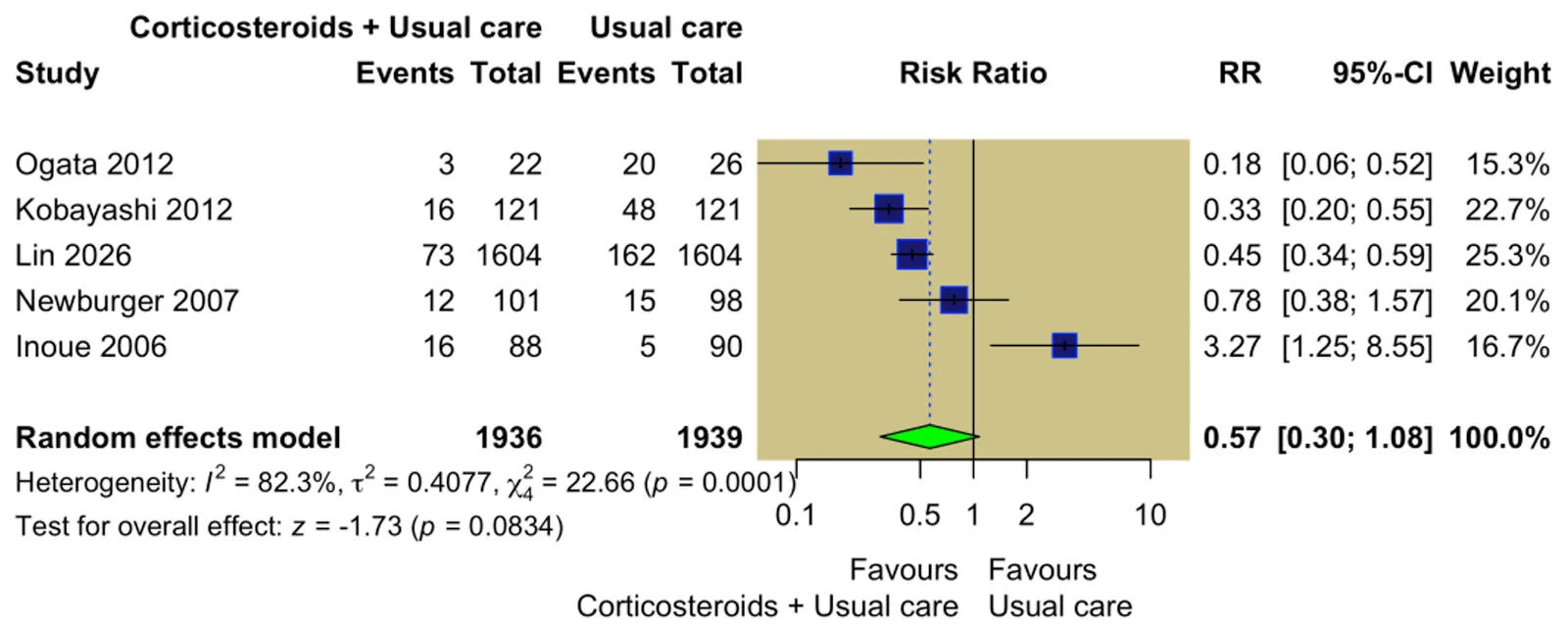
Forest plot of intravenous immunoglobulin resistance with adjunctive corticosteroid therapy versus usual care. RR: risk ratio; CI: confidence interval.

### Inflammatory and laboratory markers

Serum albumin did not differ significantly between adjunctive corticosteroid therapy plus usual care and usual care alone (MD, 0.13; 95% CI, −0.16 to 0.43; P = 0.382), with substantial heterogeneity (I^2^ = 73.0%, P = 0.024) (Fig. 7). In contrast, corticosteroid use was associated with a greater reduction in white blood cell count (MD, −1.07; 95% CI, −1.98 to −0.17; P = 0.020), with no observed heterogeneity (I^2^ = 0.0%, P = 0.887) (Fig. 8).

**Figure 7:**
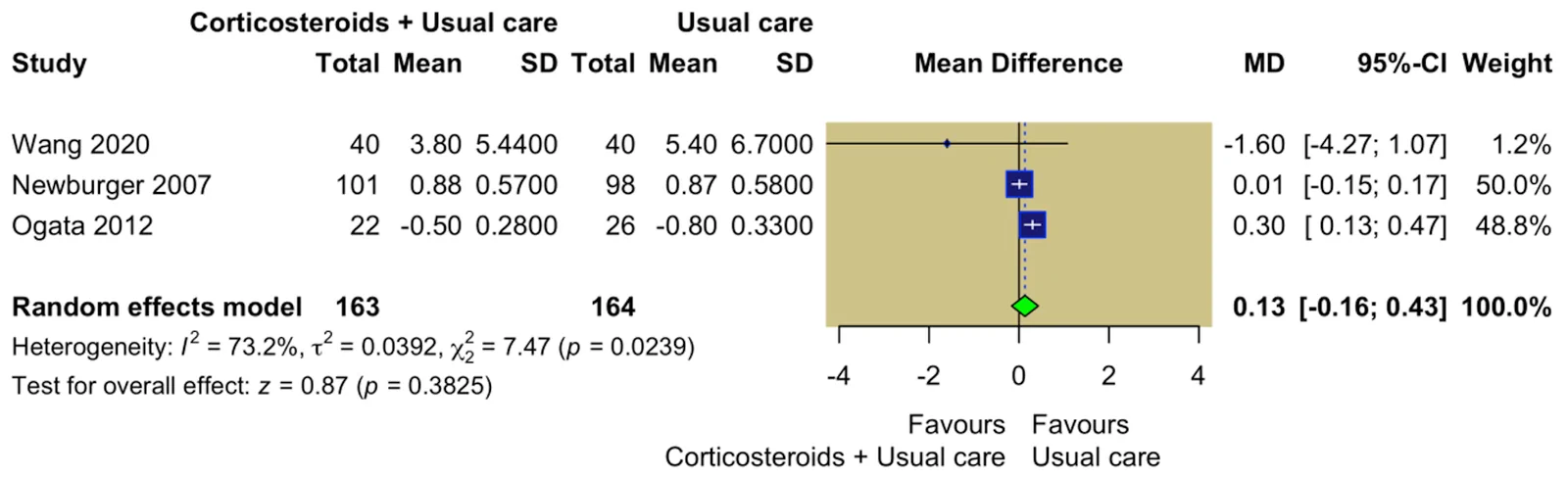
Forest plot of serum albumin with adjunctive corticosteroid therapy versus usual care.

**Figure 8:**
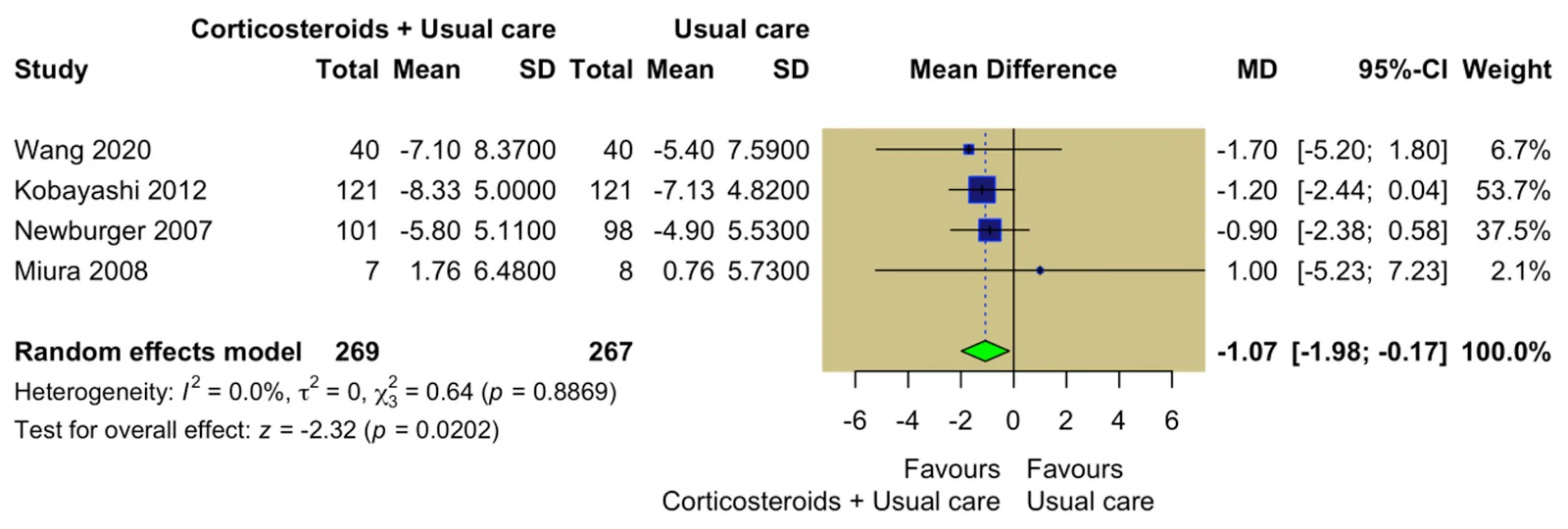
Forest plot of white blood cell counts with adjunctive corticosteroid therapy versus usual care.

For platelet count, no significant difference was observed between groups (MD, 30.87; 95% CI, −33.33 to 95.06; P = 0.346), with moderate heterogeneity (I^2^ = 56.7%, P = 0.099) (Supplementary Material 9, cr.elmerpub.com).

### Coronary and safety outcomes

For safety outcomes, adjunctive corticosteroid therapy was not associated with a significant difference in adverse events leading to discontinuation (RR, 1.34; 95% CI, 0.56 to 3.16; P = 0.510; I^2^ = 18.1%, P = 0.295) or any adverse event (RR, 0.92; 95% CI, 0.57 to 1.48; P = 0.724; I^2^ = 40.2%, P = 0.138) compared with usual care alone, Safety profiles varied across regimens (Supplementary Material 2, cr.elmerpub.com), with pulse methylprednisolone trials reporting transient events such as bradycardia, hyperglycemia, and hypertension, whereas longer prednisolone regimens showed different adverse-event patterns. Thus, the significant improvement in CRP was not accompanied by a statistically significant pooled improvement in coronary outcomes. These estimates were imprecise, particularly for coronary artery lesions, which showed substantial heterogeneity. (Supplementary Material 9, cr.elmerpub.com).

For coronary outcomes, no significant difference was observed in coronary artery lesions (RR, 0.84; 95% CI, 0.42 to 1.68; P = 0.625; I^2^ = 69.9%, P = 0.019) or large/giant aneurysms (RR, 1.55; 95% CI, 0.53 to 4.55; P = 0.427; I^2^ = 0.0%, P = 0.574) (Supplementary Material 9, cr.elmerpub.com).

## Discussion

This meta-analysis of 5,796 children summarizes randomized evidence on adjunctive corticosteroid therapy in Kawasaki disease, addressing its effects on inflammatory response, clinical recovery, coronary outcomes, and safety. Adjunctive corticosteroid therapy plus usual care was associated with a significantly greater reduction in CRP levels compared with usual care alone (MD, −11.05, P = 0.0027). The CRP finding should be interpreted primarily as evidence of enhanced suppression of systemic inflammation rather than as a surrogate for coronary protection. CRP was prespecified as the primary outcome because it offered a consistently reported quantitative measure of early inflammatory response across randomized trials; however, it is not itself a patient-important clinical endpoint. Importantly, the pooled coronary analyses did not demonstrate a statistically significant overall benefit, indicating that greater CRP reduction should not be assumed to translate directly into improved coronary outcomes. Sensitivity analysis confirmed that this finding was not driven by a single study. Corticosteroid therapy also reduced fever duration (MD, −1.03, P = 0.0025). IVIG resistance was lower with corticosteroids but did not reach statistical significance. Among laboratory outcomes, white blood cell count decreased significantly, whereas albumin and platelet count did not differ between groups. No significant differences were observed in coronary artery lesions, large/giant aneurysms, any adverse events, or adverse events leading to discontinuation. Clinical setting was an important source of heterogeneity. Evidence from high-risk initial-treatment trials should be distinguished from rescue trials in established IVIG-resistant disease, in which additional IVIG was an active comparator.

The present meta-analysis provides an updated synthesis of randomized evidence evaluating adjunctive corticosteroid therapy in children with Kawasaki disease, with the clearest efficacy signal observed for inflammatory and early clinical response outcomes. Adjunctive corticosteroid therapy was associated with a significantly greater reduction in CRP levels compared with usual care alone, and this finding was directionally stable in leave-one-out sensitivity analysis. This result is consistent with the large Lin et al [14] trial, in which reductions in CRP at 72 h were greater with prednisolone plus standard therapy than with standard therapy alone, with mean reductions of 67.5 mg/L and 59.8 mg/L, respectively. Clinical recovery also favored corticosteroid therapy, as fever duration was significantly reduced in the pooled analysis. This aligns with Lin et al [14], where rescue therapy was used in 4.6% of participants receiving prednisolone plus standard therapy compared with 10.1% receiving standard treatment alone, and median fever duration was 8.4 h versus 13.2 h, respectively. Similar clinical benefits were observed in smaller trials, including Ogata et al [22], where prompt defervescence occurred in 19 of 22 patients (86.4%) in the intravenous methylprednisolone plus IVIG group compared with six of 26 patients (23.1%) in the single-IVIG group. Sundel et al [23] also reported shorter fever duration, shorter hospital stay, lower erythrocyte sedimentation rate, and lower CRP with intravenous methylprednisolone plus aspirin/IVIG compared with aspirin/IVIG alone.

The biological plausibility of these findings is supported by the inflammatory pathogenesis of Kawasaki disease. Kawasaki disease is recognized as an immunologic disorder characterized by cytokine cascade activation and endothelial cell activation. Serum levels of multiple inflammatory cytokines, including interleukin (IL)-1β, tumor necrosis factor (TNF)-α, IL-2, IL-6, IL-8, interferon (IFN)-γ, IL-4, and IL-10, are elevated in patients with Kawasaki disease [9–11]. These cytokines may mediate endothelial injury and contribute to coronary artery disease while also producing the systemic inflammatory manifestations of the disease [11, 13]. Accordingly, corticosteroids may improve early clinical and laboratory outcomes by rapidly suppressing cytokine activity and systemic inflammation. Rapid downregulation of cytokine secretion by exogenous corticosteroids has been proposed as a mechanism that may attenuate the development of coronary artery abnormalities in Kawasaki disease [12]. This mechanism is consistent with the current meta-analysis, in which corticosteroid therapy significantly reduced CRP levels, shortened fever duration, and lowered white blood cell count, whereas albumin and platelet count were not significantly different between groups. The pattern suggests that corticosteroids may exert their most measurable effect on early inflammatory activity rather than uniformly correcting all laboratory abnormalities across different phases of the disease.

The magnitude of benefit varied across trials, likely reflecting differences in population risk, geographic setting, and corticosteroid regimen. The RAISE trial focused on Japanese patients at high risk for IVIG resistance, defined by a Kobayashi score of at least 5, and used a longer-course prednisolone regimen continued for 15 days after CRP normalization [15]. In that trial, coronary artery abnormalities were significantly lower in the IVIG plus prednisolone group than in the IVIG group, occurring in four patients (3%) versus 28 patients (23%), respectively [15]. Inoue et al [12] similarly reported fewer coronary artery abnormalities before 1 month with IVIG plus prednisolone than with IVIG alone, at 2.2% versus 11.4%. In contrast, the large trial by Lin et al [14] enrolled an unselected Kawasaki disease population and found that coronary artery lesions at 1 month occurred in 16.0% of participants receiving prednisolone plus standard treatment and 13.8% of those receiving standard treatment alone, with an adjusted risk difference of 1.1 percentage points. These contrasting findings suggest that corticosteroid efficacy may be greater in selected high-risk inflammatory phenotypes than in broad unselected populations.

Regimen differences may also explain these inconsistent treatment effects. The RAISE regimen used sustained prednisolone therapy, whereas several trials evaluated single-pulse or short-course methylprednisolone strategies [14–16]. This distinction is clinically important because the serum half-life of a pulsed dose of methylprednisolone is about 3 h, whereas prednisolone in the RAISE trial was administered over approximately 3 weeks [15]. The trial by Newburger et al [16], which tested single-pulse intravenous methylprednisolone in an unselected population, found a shorter initial hospitalization, lower erythrocyte sedimentation rate at week 1, and a tendency toward lower CRP, but did not demonstrate improved coronary outcomes. A distinct therapeutic question arises in patients with established IVIG resistance, in whom corticosteroid rescue may be considered as an alternative to an additional dose of IVIG. This comparison was directly evaluated by Miura et al (2005) [20], Miura et al (2008) [21], and Wang et al [24]. In the earlier Miura trial (2005) [20], intravenous methylprednisolone produced faster but temporary defervescence compared with additional IVIG, while adverse effects, including sinus bradycardia and hyperglycemia, were more frequently; the investigators favored additional IVIG. The later Miura trial (2008) [21] found faster but transient fever and selected cytokine suppression with methylprednisolone, without a clear between-group difference in coronary lesions. Wang et al [24] similarly compared methylprednisolone pulse therapy with a second 2 g/kg dose of IVIG. Methylprednisolone was associated with faster resolution of fever and more rapid improvement in several inflammatory markers, but treatment failure or readmission was more frequent and long-term coronary outcomes did not clearly favor the corticosteroid strategy. Therefore, additional IVIG remains an important active rescue comparator in established IVIG-resistant Kawasaki disease, and evidence from rescue therapy should be distinguished from evidence regarding corticosteroid use during initial treatment. These findings support the possibility that sustained corticosteroid exposure and tapering may be more important than brief inflammatory suppression alone.

The current results, therefore, separate early inflammatory efficacy from coronary protection. Although corticosteroids improved CRP levels and reduced fever duration, the pooled analysis did not show a significant overall reduction in coronary artery lesions or large/giant aneurysms. This finding is consistent with Lin et al [14] and Newburger et al [16], both of which failed to show a coronary benefit in unselected populations or with single-pulse therapy. However, it contrasts with the RAISE trial and the trail by Inoue et al [12, 15], where coronary outcomes favored corticosteroid-containing regimens in higher-risk or Japanese cohorts. The absence of a significant overall coronary effect may reflect heterogeneity in baseline coronary status, timing of treatment, patient risk profile, outcome definitions, and follow-up duration. It may also reflect differences in risk-score performance, as the generalizability of the RAISE regimen is limited by the poor predictive performance of the risk score and other risk scores in non-Japanese populations, making the identification of high-risk patients outside Japan challenging [15].

The pooled safety findings did not demonstrate a statistically significant difference between treatment groups, but they should be interpreted cautiously. In the pooled analysis, adjunctive corticosteroid therapy was not associated with a significant increase in any adverse event or adverse events leading to discontinuation. Individual trials nevertheless reported specific adverse effects, including blood-pressure abnormalities, bradycardia, hypothermia, hyperglycemia, hypokalemia, and infectious events. Therefore, the pooled composite safety outcomes should be interpreted alongside the trial-level adverse-event profiles. This is consistent with the study by Lin et al [14], in which the overall incidence of adverse events did not differ significantly between treatment groups. Kobayashi et al [15] also reported similar serious adverse events between groups, including high total cholesterol and neutropenia in the IVIG plus prednisolone group and high total cholesterol and non-occlusive thrombus in the IVIG group. Moreover, rare corticosteroid-related harms, including severe bacterial infection, thrombosis, bone mineral loss, osteonecrosis of the femoral head, and ophthalmic lesions, could not be fully assessed in some trials because of short follow-up and insufficient statistical power.

In the context of current guidance and prior evidence, these findings support a selective rather than universal role for corticosteroids in Kawasaki disease. Current guidelines recommend adjunctive glucocorticoids for patients at high risk for resistance to IVIG [25, 26]. The present meta-analysis is compatible with this approach, showing consistent benefit for inflammatory and fever-related outcomes but no uniform coronary benefit across all randomized populations. Prior meta-analytic evidence has also suggested benefit in treatment response, with initial treatment failure significantly lower among patients receiving corticosteroids plus IVIG than among those receiving IVIG alone [27]. Taken together, the available evidence suggests that adjunctive corticosteroids are most defensible in carefully selected high-risk or inflammation-predominant Kawasaki disease populations, while routine use in all children with Kawasaki disease remains less clearly supported.

### Strengths and limitations

This study has several strengths. It provides an updated synthesis of RCTs evaluating adjunctive corticosteroid therapy in Kawasaki disease, including large contemporary evidence and trials conducted across different clinical settings. The analysis assessed multiple clinically relevant domains, including inflammatory response, fever duration, IVIG resistance, coronary outcomes, and safety. The use of leave-one-out sensitivity analysis, Galbraith plot assessment, and meta-regression further strengthened the evaluation of robustness and potential sources of between-study variability.

However, several limitations should be considered. The included trials differed in population risk profile, corticosteroid regimen, timing of treatment, comparator therapy, follow-up duration, and outcome definitions. Some trials enrolled unselected patients, whereas others focused on high-risk or IVIG-resistant Kawasaki disease, limiting direct comparability. Substantial heterogeneity was observed for fever duration, IVIG resistance, and coronary artery lesions. In addition, several outcomes were informed by a small number of studies, and rare adverse events may have been underdetected because of limited sample size or short follow-up in some trials. In addition, definitions and ascertainment methods for adverse events varied across the included trials, which should be considered when interpreting the pooled safety estimates. Study-level meta-regression was exploratory and should be interpreted cautiously. Differences between unselected, predicted high-risk, and established IVIG-resistant populations limited direct comparability across trials.

### Clinical implications and future directions

The findings suggest that adjunctive corticosteroids may accelerate inflammatory and early clinical recovery, as reflected by greater CRP reduction and shorter fever duration. However, CRP is a surrogate inflammatory marker, and its improvement should not be interpreted as evidence of coronary protection. However, the absence of a significant overall reduction in coronary outcomes does not support routine universal corticosteroid use for all patients. A risk-stratified approach appears more appropriate, especially for children at high risk of IVIG resistance or with marked inflammatory activity. For patients with established IVIG resistance, however, the treatment question differs from that of initial adjunctive therapy. In this setting, additional IVIG represents an active rescue option and has been directly compared with methylprednisolone pulse therapy in randomized trials. The limited evidence suggests trade-offs between faster inflammatory control with corticosteroids and treatment failure or adverse effects, and does not establish a uniformly preferable rescue strategy across all outcomes.

Future trials should clarify which patients benefit most, which corticosteroid regimen is optimal, and whether prolonged tapering provides greater benefit than pulse therapy alone. Further studies should use standardized definitions for IVIG resistance and coronary outcomes, include diverse populations, and provide longer follow-up to evaluate coronary sequelae and rare adverse events.

### Conclusions

Adjunctive corticosteroid therapy in Kawasaki disease was associated with greater suppression of systemic inflammation and shorter fever duration. However, the improvement in CRP, a surrogate inflammatory marker, was not accompanied by a statistically significant overall reduction in coronary outcomes. No statistically significant difference in the pooled adverse-event outcomes was detected; however, this does not exclude uncommon or delayed harms. These findings support selective, risk-based use rather than routine universal administration, with further trials needed to define the optimal population and regimen. In established IVIG-resistant disease, corticosteroid rescue should be interpreted separately from initial adjunctive therapy because additional IVIG represents an active alternative rescue strategy.

## Supplementary Material

Suppl 1 Electronic search strategy.

Suppl 2 Definitions of safety outcomes included in the quantitative synthesis.

Suppl 3 Summary characteristics of included studies.

Suppl 4 Galbraith plot for change in C-reactive protein levels.

Suppl 5 Meta-regression of change in C-reactive protein levels by mean age.

Suppl 6 Meta-regression of change in C-reactive protein levels by male proportion.

Suppl 7 Doi plot and LFK index for change in C-reactive protein levels.

Suppl 8 Funnel plot for change in C-reactive protein levels.

Suppl 9 Summary of meta-analytic findings for adjunctive corticosteroid therapy versus usual care.

## Data Availability

All data used in this systematic review and meta-analysis were extracted from published studies. The extracted dataset is available from the corresponding author upon reasonable request.
