# Supplementary material for: Efficacy and Safety of Adjunctive Corticosteroids in Children With Kawasaki Disease: A Systematic Review and Meta-Analysis of Randomized Trials
Source: Cardiol Res. 2026 Oct 24;17(6):616–29. doi: 10.14740/cr2300 (PMC13719651; doi:10.14740/cr2300)
Supplement: Supplementary file 1 [file cr-17-06-616-s001.docx]

**Suppl 1.** Search strategy for included studies

| **Databases** | **Search Query** | **Filters** | **Results** |
| --- | --- | --- | --- |
| **PubMed** | ("Kawasaki disease" OR KD) AND (corticosteroid* OR steroid* OR prednisolone OR methylprednisolone OR glucocorticoid* OR IVMP) AND (IVIG OR "intravenous immunoglobulin") AND (coronary OR aneurysm OR lesion* OR refractory OR resistant OR fever) | All Filters | 653 |
| **Scopus** | ("Kawasaki disease" OR KD) AND (corticosteroid* OR steroid* OR prednisolone OR methylprednisolone OR glucocorticoid* OR IVMP) AND (IVIG OR "intravenous immunoglobulin") AND (coronary OR aneurysm OR lesion* OR refractory OR resistant OR fever) | Title, Abstract, and Keywords | 925 |
| **Wos** | ("Kawasaki disease" OR KD) AND (corticosteroid* OR steroid* OR prednisolone OR methylprednisolone OR glucocorticoid* OR IVMP) AND (IVIG OR "intravenous immunoglobulin") AND (coronary OR aneurysm OR lesion* OR refractory OR resistant OR fever) | All Filters | 519 |
| **Cochrane** | ("Kawasaki disease" OR KD) AND (corticosteroid* OR steroid* OR prednisolone OR methylprednisolone OR glucocorticoid* OR IVMP) AND (IVIG OR "intravenous immunoglobulin") AND (coronary OR aneurysm OR lesion* OR refractory OR resistant OR fever) | Title, Abstract, and Keywords | 54 |
