# Supplementary material for: Efficacy and Safety of Adjunctive Corticosteroids in Children With Kawasaki Disease: A Systematic Review and Meta-Analysis of Randomized Trials
Source: Cardiol Res. 2026 Oct 24;17(6):616–29. doi: 10.14740/cr2300 (PMC13719651; doi:10.14740/cr2300)
Supplement: Supplementary file 2 [file cr-17-06-616-s002.docx]

**Suppl 2.** Definitions of safety outcomes included in the quantitative synthesis

| **Outcome** | **Study** | **Trial-specific definition** |
| --- | --- | --- |
| **Any adverse event** | Sundel et al., 2003 | Occurrence of ≥1 prospectively defined adverse event during hospitalization; events were documented through daily medical-record review. |
|  | Lin et al., 2026 | NA; reported as “any adverse event,” without an explicit operational composite definition in the main article. |
|  | Newburger et al., 2007 | Occurrence of ≥1 adverse event; events were classified by severity, expectedness, and attributability and centrally adjudicated. |
|  | Okada et al., 2003 | NA; only absence of adverse reactions to steroids was reported. |
|  | Kobayashi et al., 2012 | Not applicable to this outcome; reported data represent serious adverse events rather than any adverse event. |
|  | Ogata et al., 2012 | Occurrence of prospectively defined adverse events documented by daily medical-record review; hypothermia <36°C, bradycardia <60 beats/min, hypertension SBP ≥120 mmHg. |
| **Adverse events leading to treatment discontinuation** | Lin et al., 2026 | Any adverse event leading to discontinuation or adjustment of the treatment dose. |
|  | Inoue et al., 2006 | Discontinuation of protocol treatment because of an adverse event. |
|  | Ogata et al., 2012 | NA; treatment discontinuation because of adverse events was not reported. |
