# Supplementary material for: Efficacy and Safety of Adjunctive Corticosteroids in Children With Kawasaki Disease: A Systematic Review and Meta-Analysis of Randomized Trials
Source: Cardiol Res. 2026 Oct 24;17(6):616–29. doi: 10.14740/cr2300 (PMC13719651; doi:10.14740/cr2300)
Supplement: Supplementary file 3 [file cr-17-06-616-s003.docx]

Suppl 3. Summary characteristics of included studies

| Study | Arms | Sample size | Country | Study design | Trial, or database, or center name | Recruitment | Population type | Baseline coronary artery status (CAA yes/no) | Definition of IVIG resistance | Follow-up (months) | Inclusion criteria | Conclusion |
| --- | --- | --- | --- | --- | --- | --- | --- | --- | --- | --- | --- | --- |
| Lin et al, 2026 [14] | Standard Tx + corticosteroids | 3,208 | China | RCT | Chinese Kawasaki Disease Collaboration Network (28 sites); NCT04078568 | January 2020 – November 2024 | Unselected newly diagnosed KD | Yes (mixed) | Persistent or recurrent fever (axillary ≥ 37.5 °C or rectal ≥ 38 °C) at 36 h after completion of the initial IVIG infusion | 3 | Children ≥ 1 month old meeting 2017 AHA KD criteria, diagnosed within 10 days of fever onset, with no prior IVIG treatment | This phase 3 randomized trial investigated the addition of adjunctive prednisolone to standard IVIG and aspirin therapy in unselected Chinese children with KD. The results demonstrated no significant reduction in the primary endpoint of coronary artery lesions at 1 month, which occurred in 16.0% of the prednisolone arm vs. 13.8% of the standard therapy arm. However, adjunctive prednisolone did yield significant secondary anti-inflammatory benefits, including a shorter median duration of fever (8.4 vs. 13.2 h) and a lower requirement for subsequent rescue therapy (4.6% vs. 10.1%) compared to the standard treatment cohort. The overall incidence of adverse events remained comparable between the two groups. Ultimately, despite accelerating clinical recovery and fever resolution, the routine addition of systemic glucocorticoids does not confer superior coronary protection over standard primary therapy in general populations. |
|  | Standard Tx | 1,604 |  |  |  |  |  |  |  |  |  |  |
| Kobayashi et al, 2012 [15] | Standard Tx + corticosteroids | 248 | Japan | RCT | RAISE study group (74 hospitals); UMIN000000940 | September 2008 – December 2010 | High-risk/severe KD predicted to be IVIG-resistant | No | Axillary temperature ≥ 37.5 °C lasting more than 24 h after completion of primary treatment | 1 | Japanese patients meeting KD diagnostic guidelines with a risk score ≥ 5 points (predictive of IVIG non-response). Excluded those with illness duration ≥9 days, baseline CAA, prior KD history, recent corticosteroid/IVIG use, or severe comorbidities. | This multicenter RCT evaluated the efficacy of adding a prolonged course of prednisolone to standard IVIG and aspirin therapy in Japanese children with severe, high-risk KD. The intervention significantly reduced the primary endpoint, the incidence of CAA, which occurred in only 3% of the prednisolone group compared to 23% in the standard therapy group. Furthermore, patients receiving adjunctive prednisolone experienced more rapid fever resolution, quicker normalization of inflammatory markers such as CRP, and a markedly reduced need for secondary rescue treatments. Both study arms demonstrated a similar, low incidence of serious adverse events, including isolated cases of hypercholesterolemia and neutropenia; however, the study lacked sufficient power to detect extremely rare events. Overall, these findings strongly support the early addition of corticosteroids to standard therapy to improve coronary outcomes in high-risk KD populations. |
|  | Standard Tx | 123 |  |  |  |  |  |  |  |  |  |  |
| Ogata et al, 2012 [22] | Standard Tx + corticosteroids | 22 | Japan | RCT | Kitasato University Hospital; UMIN000005021 | April 2007 – November 2010 | Predicted IVIG-resistant KD | No | Persistent or recrudescent fever (axillary temperature ≥ 37.5 °C) at 36 h after the completion of the initial treatment | 1 | Japanese patients fulfilling standard diagnostic guidelines for acute KD. Patients had to be predicted as high-risk/refractory using the Egami score. Excluded those with a prior history of KD, baseline coronary artery lesions, or prior steroid treatment. | This prospective randomized trial evaluated the efficacy and safety of adding IV MPT to standard IVIG and aspirin for KD patients predicted to be refractory to initial treatment. The intervention arm demonstrated significantly superior clinical effectiveness, including a higher prompt defervescence rate (86.4% vs. 23.1%), a shorter total duration of fever, and faster normalization of systemic inflammatory markers like CRP compared to the IVIG-alone group. Crucially, the adjunct steroid pulse therapy resulted in significantly smaller coronary artery dimension z-scores at 1 month, indicating stronger protection against coronary lesions. While transient and minor adverse events such as hypothermia and bradycardia were more frequently observed in the steroid group, no serious adverse events occurred. Ultimately, the early combined use of steroid pulse therapy and IVIG provides safe and highly effective primary treatment for high-risk KD populations. |
|  | Standard Tx | 26 |  |  |  |  |  |  |  |  |  |  |
| Newburger et al, 2007 [16] | Standard Tx + corticosteroids | 101 | North America (USA and Canada) | RCT | Pediatric Heart Network (8 centers); NCT00132080 | December 2002 – December 2004 | General/unselected KD | Yes (mixed) | Temperature of ≥ 38.3 °C occurring 36 h or more after the completion of the initial IVIG infusion | 1.25 | Patients on days 4 to 10 of illness meeting standard diagnostic criteria for KD or demonstrating baseline coronary abnormalities/aneurysms along with partial clinical criteria. Excluded patients with prior IVIG therapy or recent systemic corticosteroid use. | This double-blind RCT investigated whether adding a single pulsed dose of IV methylprednisolone to standard IVIG and aspirin therapy improved outcomes in unselected children with KD. The addition of the corticosteroid pulse did not significantly decrease the prevalence or severity of CAA at 1 or 5 weeks post-randomization. While the intervention group experienced a marginally shorter initial hospitalization and faster early reductions in inflammatory markers such as ESR and CRP, the overall total duration of fever, length of hospital stay, and requirement for IVIG retreatment were similar between the two groups. Adverse events occurred at comparable rates, with no serious events directly attributed to the study drug. Thus, routine primary use of pulsed methylprednisolone is not recommended for general KD populations; however, *post hoc* analysis suggested a potential coronary benefit among patients who ultimately proved resistant to initial IVIG therapy. |
|  | Standard Tx | 98 |  |  |  |  |  |  |  |  |  |  |
| Inoue et al, 2006 [12] | Standard Tx + corticosteroids | 90 | Japan | RCT | Gunma Kawasaki Disease Study Group (12 medical institutions) | September 2000 – March 2005 | General acute KD | No | Persistent fever lasting more than 24 h after completion of the 2 IVIG infusions, or recrudescent fever associated with KD symptoms after an afebrile period | 50 | Patients fulfilling clinical diagnostic criteria for KD (at least 5 standard symptoms including fever). Excluded patients with a prior history of KD, baseline CAA, or initial consultation after 9 days from illness onset | This multicenter, randomized trial evaluated the addition of corticosteroids to primary IVIG therapy in unselected children with acute KD. The combined IVIG and prednisolone regimen demonstrated significant clinical superiority, substantially reducing the incidence of CAA within 1 month (2.2% vs. 11.4%) compared to standard IVIG therapy. Furthermore, the intervention cohort experienced significantly faster fever resolution, quicker normalization of CRP levels, and lower rates of initial treatment failure. Both treatments were well tolerated, with no major side effects directly attributed to corticosteroid use; however, minor isolated events such as transient inability to stand and gastroenteritis were observed. Ultimately, these findings strongly support the early use of combined corticosteroid and IVIG therapy to improve coronary outcomes and accelerate clinical recovery in general KD populations. |
|  | Standard Tx | 88 |  |  |  |  |  |  |  |  |  |  |
| Sundel et al, 2003 [23] | Standard Tx + corticosteroids | 18 | USA | RCT | Children's Hospital, Boston | February 1998 – November 2000 | General/unselected KD | Yes (mixed) | Temperature of ≥ 38.3 °C occurring more than 48 h after the completion of the initial IVIG treatment, without an alternative explanation | 1.5 | Children with fever for ≤ 10 days who met at least four of the five clinical criteria for KD. Excluded those with a prior history of KD, baseline coronary aneurysms (by Japanese criteria), possible infections, or contraindications to steroids. | This prospective randomized trial evaluated the efficacy of adding a single pulse of IV methylprednisolone (IVMP) to standard initial IVIG and aspirin therapy in a general population of children with acute KD. The intervention arm demonstrated significantly faster clinical recovery, characterized by a shorter total duration of fever and decreased hospital length of stay compared to the standard therapy group. Furthermore, patients receiving IVMP showed more rapid normalization of systemic inflammatory markers, specifically CRP and ESR, by 6 weeks. While the study lacked sufficient statistical power to detect major differences in long-term coronary artery outcomes, the incidence of coronary abnormalities remained similar between the two groups. The adjunct steroid therapy was well tolerated, with infrequent adverse events, indicating that pulse methylprednisolone is a safe and effective approach for accelerating systemic anti-inflammatory recovery in acute KD. |
|  | Standard Tx | 21 |  |  |  |  |  |  |  |  |  |  |
| Wang et al, 2020 [24] | IV methylprednisolone 15 mg/kg/day for 3 days, without subsequent oral prednisone taper | 40 | China | RCT | Guangzhou Women and Children’s Medical Center; ChiCTR-EOC-17013266 | January 2018 – Junn 2019 | Established IVIG-resistant KD | Yes (mixed) | Recrudescent or persistent fever at least 36 h after the end of the first IVIG infusion | 24 | Hospitalized patients meeting 2017 AHA KD diagnostic criteria who demonstrated resistance to initial IVIG therapy. Excluded patients with prior KD history, recent corticosteroid/immunosuppressant use, or other conditions affecting temperature. | This prospective RCT investigated the efficacy of MPT versus a second dose of IVIG in children with IVIG-resistant KD. In the short term, MPT demonstrated significantly lower medical costs, faster fever resolution, and more rapid normalization of inflammatory markers (CRP, neutrophils, platelets) without an increased short-term risk of coronary artery lesions. However, MPT was associated with a significantly higher treatment failure and readmission rate (27.5% vs. 10.0%), often requiring third-line rescue therapy. Crucially, the MPT group experienced a higher incidence of coronary artery lesions during the long-term follow-up at 12 and 24 months. Regarding safety, transient bradycardia occurred in five MPT patients but resolved spontaneously, while the IVIG group reported no adverse events. Ultimately, despite early anti-inflammatory benefits, caution is strongly advised against the routine use of MPT without a subsequent oral taper for IVIG-resistant KD due to inferior long-term coronary outcomes and elevated readmission rates. |
|  | Additional IVIG 2 g/kg, single infusion | 40 |  |  |  |  |  |  |  |  |  |  |
| Miura et al, 2008 [21] | IV methylprednisolone 30 mg/kg/day for 3 days | 7 | Japan | RCT | Tokyo Metropolitan Kiyose Children’s Hospital | January 2001 – May 2003 | Established IVIG-resistant KD | NA | Failure to become afebrile after an initial single infusion of IVIG (2 g/kg over 24 h) | 0.25 | Patients with acute KD who failed to respond to an initial primary treatment of 2 g/kg IVIG infused over a 24-h period | This prospective randomized study evaluated the clinical effects and cytokine profiles of IVMP therapy compared to a second dose of IVIG in children with IVIG-resistant KD. The findings revealed that IVMP induced a significantly faster initial suppression of fever and key inflammatory cytokines, particularly MCP-1 and TNF-α. However, these clinical and anti-inflammatory benefits were strictly transient; patients in the IVMP group subsequently experienced recurrent fevers and a rebound in cytokine levels, ultimately matching the IVIG group by day 4. Furthermore, the overall prevalence of coronary artery lesions was similar between both treatment arms, and IVMP patients experienced a significantly higher rate of adverse events, specifically bradycardia and hyperglycemia. Ultimately, due to the cytokine rebound effect and the lack of superior long-term coronary protection, the use of IVMP without a subsequent oral steroid taper is not recommended for initial IVIG resistance. |
|  | Additional IVIG 2 g/kg | 8 |  |  |  |  |  |  |  |  |  |  |
| Miura et al, 2005 [20] | Standard Tx + corticosteroids | 11 | Japan | RCT | Tokyo Metropolitan Kiyose Children’s Hospital | NA | Established IVIG-resistant KD | NA | Persistent or recurrent fever (> 37.5 °C) 48 h after a single initial infusion of IVIG (2 g/kg) | 0.25 | KD patients demonstrating persistent or recurrent fever (> 37.5 °C) 48 h after receiving an initial single infusion of 2 g/kg IVIG | This prematurely halted RCT evaluated the safety and efficacy of IVMP therapy vs. a second dose of IVIG in children with IVIG-resistant KD. The findings demonstrated that while IVMP provided a significantly faster antipyretic effect during the first 2 days, this benefit was strictly temporary, with fever recurrence rates equalizing between the arms by day 4. Coronary artery outcomes and lesion prevalence remained similar between the two treatment groups. Crucially, the study was halted early because the IVMP cohort experienced a significantly higher incidence of transient adverse events, particularly sinus bradycardia, hypothermia, and hyperglycemia. Because IVMP resulted in higher adverse effect rates and only temporary fever resolution without superior coronary protection, the authors strongly advise that patients refractory to initial IVIG should be treated with additional IVIG rather than pulse steroids. |
|  | Standard Tx | 11 |  |  |  |  |  |  |  |  |  |  |
| Okada et al, 2003 [13] | Standard Tx + corticosteroids | 14 | Japan | RCT | Gunma Kawasaki Disease Study Group (9 medical institutes) | June 2001 – May 2002 | General acute KD | No | NA | 1 | Patients fulfilling standard diagnostic criteria for KD (at least five typical symptoms) who were enrolled within 9 days of illness onset (defining the first day of fever as day 1) | This multicenter RCT evaluated the efficacy of adding corticosteroids to standard IV gamma globulin (IVGG) therapy for children in the acute phase of KD. The combination therapy demonstrated significantly faster clinical recovery, evidenced by a much shorter duration of fever (0.3 vs. 2.9 days) and a more rapid normalization of CRP levels compared to the standard IVGG-alone group. Furthermore, adjunctive corticosteroids successfully induced a rapid and significant reduction in circulating inflammatory cytokines (such as IL-2, IL-6, IL-8, and IL-10) within 24 h of administration—an anti-inflammatory effect that IVGG alone failed to achieve. The safety profile was favorable, with no adverse reactions attributed to the steroid treatment, and no patients in either cohort developed CAA. Ultimately, these findings indicate that the early addition of corticosteroids safely downregulates systemic cytokine cascades and accelerates clinical improvement in general KD populations. |
|  | Standard Tx | 18 |  |  |  |  |  |  |  |  |  |  |

IVIG: intravenous immunoglobulin; KD: Kawasaki disease; RCT: randomized controlled trial; Tx: treatment; CAA: coronary artery abnormalities; IVMP: intravenous methylprednisolone pulse; MPT: methylprednisolone pulse therapy; CRP: C-reactive protein; ESR: erythrocyte sedimentation rate; AHA: American Heart Association; IL: interleukin; TNF-α: tumor necrosis factor alpha; MCP-1: monocyte chemoattractant protein-1; NA: not available/not applicable; IV: intravenous.

References

1.	McCrindle BW, Rowley AH, Newburger JW, Burns JC, Bolger AF, Gewitz M, Baker AL, et al. Diagnosis, treatment, and long-term management of Kawasaki disease: a scientific statement for health professionals from the American Heart Association. Circulation. 2017;135(17):e927-e999.
doi:10.1161/CIR.0000000000000484
PubMed PMID:28356445

2.	Nakamura Y, Yashiro M, Yanagawa H. Epidemiology of Kawasaki disease in Japan in 2021-2022: Results of the 27th nationwide survey. Pediatr Int. 2025;67(1):e70007.
doi:10.1111/ped.70007
PubMed PMID:40189891

3.	Daniels LB, Tjajadi MS, Walford HH, Jimenez-Fernandez S, Trofimenko V, Fick DB, Jr., Phan HA, et al. Prevalence of Kawasaki disease in young adults with suspected myocardial ischemia. Circulation. 2012;125(20):2447-2453.
doi:10.1161/CIRCULATIONAHA.111.082107
PubMed PMID:22595319

4.	The Pediatric Infectious Disease Journal, n.d. https://journals.lww.com/pidj/abstract/1995/03000/Evaluation_of_the_cytokine_response_in_Kawasaki.6.aspx (accessed May 26, 2026).

5.	Furusho K, Kamiya T, Nakano H, Kiyosawa N, Shinomiya K, Hayashidera T, Tamura T, et al. High-dose intravenous gammaglobulin for Kawasaki disease. Lancet. 1984;2(8411):1055-1058.
doi:10.1016/s0140-6736(84)91504-6
PubMed PMID:6209513

6.	Xie LP, Yan WL, Huang M, Huang MR, Chen S, Huang GY, Liu F. Epidemiologic features of Kawasaki disease in Shanghai from 2013 through 2017. J Epidemiol. 2020;30(10):429-435.
doi:10.2188/jea.JE20190065
PubMed PMID:31548437

7.	Kim GB, Eun LY, Han JW, Kim SH, Yoon KL, Han MY, Yu JJ, et al. Epidemiology of Kawasaki disease in South Korea: a nationwide survey 2015-2017. Pediatr Infect Dis J. 2020;39(11):1012-1016.
doi:10.1097/INF.0000000000002793
PubMed PMID:33075217

8.	Narayan HK, Lizcano A, Lam-Hine T, Ulloa-Gutierrez R, Bainto EV, Garrido-Garcia LM, Estripeaut D, et al. Clinical Presentation and Outcomes of Kawasaki Disease in Children from Latin America: A Multicenter Observational Study from the REKAMLATINA Network. J Pediatr. 2023;263:113346.
doi:10.1016/j.jpeds.2023.02.001
PubMed PMID:36775190

9.	Furukawa S, Matsubara T, Jujoh K, Yone K, Sugawara T, Sasai K, Kato H, et al. Peripheral blood monocyte/macrophages and serum tumor necrosis factor in Kawasaki disease. Clin Immunol Immunopathol. 1988;48(2):247-251.
doi:10.1016/0090-1229(88)90088-8
PubMed PMID:3390972

10.	Matsubara T, Furukawa S, Yabuta K. Serum levels of tumor necrosis factor, interleukin 2 receptor, and interferon-gamma in Kawasaki disease involved coronary-artery lesions. Clin Immunol Immunopathol. 1990;56(1):29-36.
doi:10.1016/0090-1229(90)90166-n
PubMed PMID:2113446

11.	Mahmoudinezhad Dezfouli SM, Salehi S, Khosravi S. Pathogenic and therapeutic roles of cytokines in Kawasaki diseases. Clin Chim Acta. 2022;532:21-28.
doi:10.1016/j.cca.2022.05.015
PubMed PMID:35609708

12.	Inoue Y, Okada Y, Shinohara M, Kobayashi T, Kobayashi T, Tomomasa T, Takeuchi K, et al. A multicenter prospective randomized trial of corticosteroids in primary therapy for Kawasaki disease: clinical course and coronary artery outcome. J Pediatr. 2006;149(3):336-341.
doi:10.1016/j.jpeds.2006.05.025
PubMed PMID:16939743

13.	Okada Y, Shinohara M, Kobayashi T, Inoue Y, Tomomasa T, Kobayashi T, Morikawa A, et al. Effect of corticosteroids in addition to intravenous gamma globulin therapy on serum cytokine levels in the acute phase of Kawasaki disease in children. J Pediatr. 2003;143(3):363-367.
doi:10.1067/s0022-3476(03)00387-1
PubMed PMID:14517521

14.	Lin S, He Y, He L, Liu Y, Wang F, Xiong Z, Lin Y, et al. Randomized trial of adjunctive prednisolone for Kawasaki disease. N Engl J Med. 2026;394(15):1480-1490.
doi:10.1056/NEJMoa2511478
PubMed PMID:41985133

15.	Kobayashi T, Saji T, Otani T, Takeuchi K, Nakamura T, Arakawa H, Kato T, et al. Efficacy of immunoglobulin plus prednisolone for prevention of coronary artery abnormalities in severe Kawasaki disease (RAISE study): a randomised, open-label, blinded-endpoints trial. Lancet. 2012;379(9826):1613-1620.
doi:10.1016/S0140-6736(11)61930-2
PubMed PMID:22405251

16.	Newburger JW, Sleeper LA, McCrindle BW, Minich LL, Gersony W, Vetter VL, Atz AM, et al. Randomized trial of pulsed corticosteroid therapy for primary treatment of Kawasaki disease. N Engl J Med. 2007;356(7):663-675.
doi:10.1056/NEJMoa061235
PubMed PMID:17301297

17.	Page MJ, McKenzie JE, Bossuyt PM, Boutron I, Hoffmann TC, Mulrow CD, Shamseer L, et al. The PRISMA 2020 statement: an updated guideline for reporting systematic reviews. BMJ. 2021;372:n71.
doi:10.1136/bmj.n71
PubMed PMID:33782057

18.	Cochrane Handbook for Systematic Reviews of Interventions n.d. https://training.cochrane.org/handbook (accessed April 6, 2025).

19.	RoB 2: A revised Cochrane risk-of-bias tool for randomized trials | Cochrane Bias n.d. https://methods.cochrane.org/bias/resources/rob-2-revised-cochrane-risk-bias-tool-randomized-trials (accessed April 21, 2025).

20.	Miura M, Ohki H, Yoshiba S, Ueda H, Sugaya A, Satoh M, Yamagishi H. Adverse effects of methylprednisolone pulse therapy in refractory Kawasaki disease. Arch Dis Child. 2005;90(10):1096-1097.
doi:10.1136/adc.2004.062299
PubMed PMID:16177169

21.	Miura M, Kohno K, Ohki H, Yoshiba S, Sugaya A, Satoh M. Effects of methylprednisolone pulse on cytokine levels in Kawasaki disease patients unresponsive to intravenous immunoglobulin. Eur J Pediatr. 2008;167(10):1119-1123.
doi:10.1007/s00431-007-0642-5
PubMed PMID:18175148

22.	Ogata S, Ogihara Y, Honda T, Kon S, Akiyama K, Ishii M. Corticosteroid pulse combination therapy for refractory Kawasaki disease: a randomized trial. Pediatrics. 2012;129(1):e17-23.
doi:10.1542/peds.2011-0148
PubMed PMID:22144699

23.	Sundel RP, Baker AL, Fulton DR, Newburger JW. Corticosteroids in the initial treatment of Kawasaki disease: report of a randomized trial. J Pediatr. 2003;142(6):611-616.
doi:10.1067/mpd.2003.191
PubMed PMID:12838187

24.	Wang Z, Chen F, Wang Y, Li W, Xie X, Liu P, Zhang X, et al. Methylprednisolone pulse therapy or additional IVIG for patients with IVIG-resistant Kawasaki disease. J Immunol Res. 2020;2020:4175821.
doi:10.1155/2020/4175821
PubMed PMID:33299898

25.	Jone PN, Tremoulet A, Choueiter N, Dominguez SR, Harahsheh AS, Mitani Y, Zimmerman M, et al. Update on diagnosis and management of Kawasaki disease: a scientific statement from the American Heart Association. Circulation. 2024;150(23):e481-e500.
doi:10.1161/CIR.0000000000001295
PubMed PMID:39534969

26.	Research Committee of the Japanese Society of Pediatric Cardiology, Cardiac Surgery Committee for Development of Guidelines for Medical Treatment of Acute Kawasaki Disease. Guidelines for medical treatment of acute Kawasaki disease: report of the Research Committee of the Japanese Society of Pediatric Cardiology and Cardiac Surgery (2012 revised version). Pediatr Int. 2014;56(2):135-158.
doi:10.1111/ped.12317
PubMed PMID:24730626

27.	Wooditch AC, Aronoff SC. Effect of initial corticosteroid therapy on coronary artery aneurysm formation in Kawasaki disease: a meta-analysis of 862 children. Pediatrics. 2005;116(4):989-995.
doi:10.1542/peds.2005-0504
PubMed PMID:16199713
