# Supplementary material for: Efficacy and Safety of Adjunctive Corticosteroids in Children With Kawasaki Disease: A Systematic Review and Meta-Analysis of Randomized Trials
Source: Cardiol Res. 2026 Oct 24;17(6):616–29. doi: 10.14740/cr2300 (PMC13719651; doi:10.14740/cr2300)
Supplement: Supplementary file 9 [file cr-17-06-616-s009.docx]

**Suppl 9. Summary of meta-analytic findings for adjunctive corticosteroid therapy versus usual care**

*Adjunctive corticosteroid therapy plus usual care versus usual care alone*

| **Outcome** | **Studies, n** | **Corticosteroids + usual care** | **Usual care** | **Effect measure** | **Pooled effect (95% CI)** | **Heterogeneity** | **Overall effect** |
| --- | --- | --- | --- | --- | --- | --- | --- |
| Platelet count | 3 | N = 163 | N = 164 | MD | 30.87 (−33.33 to 95.06) | I² = 56.7%; τ² = 1783.4292; χ² (2) = 4.62; p = 0.0994 | z = 0.94; p = 0.3460 |
| Adverse events leading to discontinuation | 3 | 18/1714 | 15/1720 | RR | 1.34 (0.56 to 3.16) | I² = 18.1%; τ² = 0.1288; χ² (2) = 2.44; p = 0.2950 | z = 0.66; p = 0.5101 |
| Any adverse event | 6 | 72/1880 | 83/1888 | RR | 0.92 (0.57 to 1.48) | I² = 40.2%; τ² = 0.1221; χ² (5) = 8.36; p = 0.1375 | z = −0.35; p = 0.7237 |
| Coronary-artery lesions | 4 | 194/1914 | 175/1913 | RR | 0.84 (0.42 to 1.68) | I² = 69.9%; τ² = 0.2873; χ² (3) = 9.96; p = 0.0189 | z = −0.49; p = 0.6251 |
| Large or giant aneurysms | 3 | 8/1813 | 5/1815 | RR | 1.55 (0.53 to 4.55) | I² = 0.0%; τ² = 0; χ² (2) = 1.11; p = 0.5736 | z = 0.80; p = 0.4265 |

**Notes:** Binary outcomes are presented as events/total; platelet count is presented as total participants. All pooled estimates were calculated using a random-effects model. CI, confidence interval; MD, mean difference; RR, risk ratio
