# Supplementary material for: Efficacy and Safety of Adjunctive Corticosteroids in Children With Kawasaki Disease: A Systematic Review and Meta-Analysis of Randomized Trials
Source: Cardiol Res. 2026 Oct 24;17(6):616–29. doi: 10.14740/cr2300 (PMC13719651; doi:10.14740/cr2300)
Supplement: Supplementary file 10 [file cr-17-06-616-s004.docx]

**Suppl 4.** Galbraith plot for change in C-reactive protein levels
